# Supplementary material for: Reconstituted cell-free protein synthesis using in vitro transcribed tRNAs
Source: Commun Biol. 2020 Jul 3;3:350. doi: 10.1038/s42003-020-1074-2 (PMC7334211; doi:10.1038/s42003-020-1074-2)
Supplement: Supplementary file 1 — Supplementary Information [file 42003_2020_1074_MOESM1_ESM.pdf]

Supplementary Figures

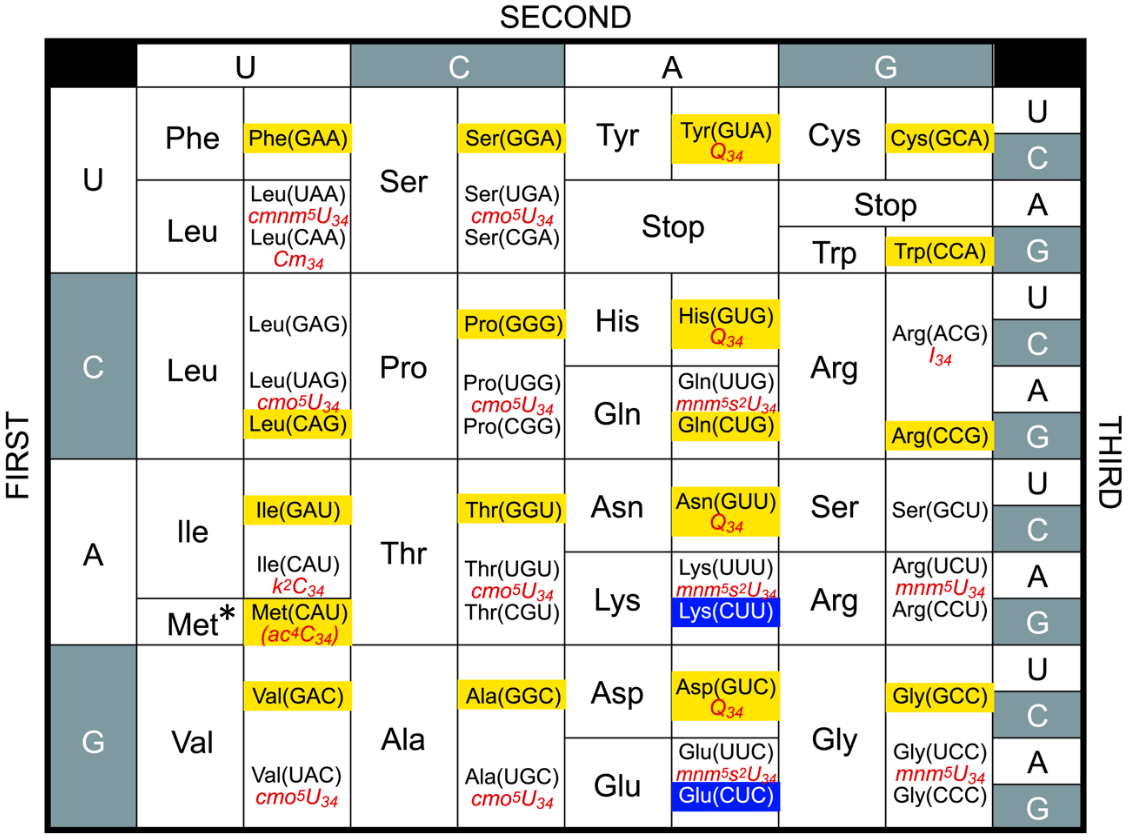

Supplementary Figure 1. Genetic code table with nucleotide modifications at the 34 position. Genetic code table showing 41 tRNA species in *E. coli*. Red characters represent nucleotide modifications at the 34 position of each tRNA. tRNAs selected for reconstitution are highlighted in yellow. Artificially designed $tRNA^{Lys}_{CUU}$ and $tRNA^{Glu}_{CUC}$ are highlighted in blue. *$tRNA^{fMet}_{CAU}$ and $tRNA^{mMet}_{CAU}$ correspond to the Met codon and ac^4C34 modification is only given in $tRNA^{mMet}_{CAU}$.

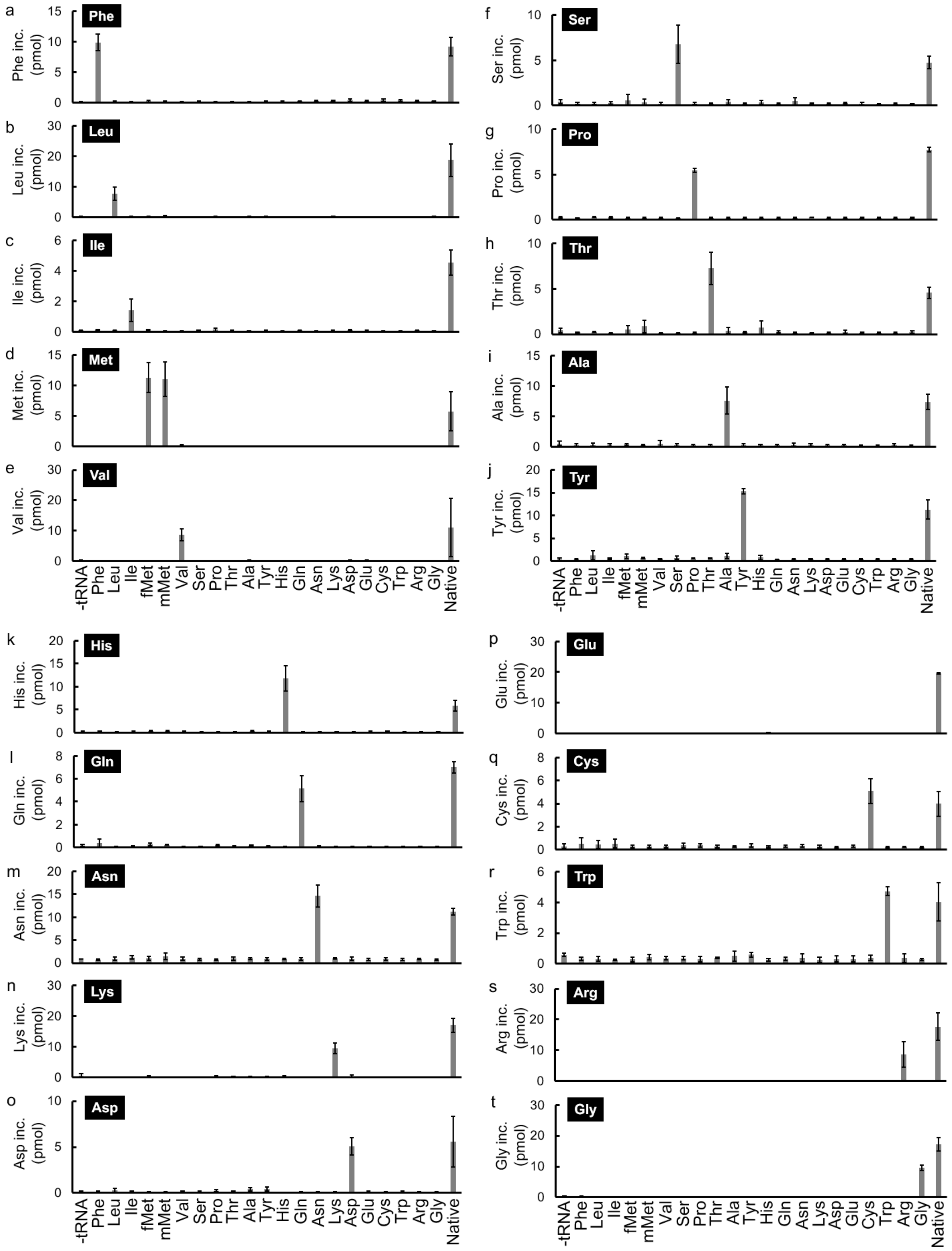

Supplementary Figure 2. Aminoacylation of iVTtRNAs. The amino acid acceptance of the prepared 21 iVTtRNAs was comprehensively analyzed for all 20 amino acids. Radioisotope-labeled (a) Phe, (b) Leu, (c) Ile, (d) Met, (e) Val, (f) Ser, (g) Pro, (h) Thr, (i) Ala, (j) Tyr, (k) His, (l) Gln, (m) Asn, (n) Lys, (o) Asp, (p) Glu, (q) Cys, (r) Trp, (s) Arg, and (t) Gly were used for each experiment. The concentration of each aaRS was 50 nM, and 2 A_{260} unit/mL of iVTtRNA (28–32 pmol in each 10 μL reaction mixture) or 40

A_{260} unit/mL of native tRNA mixtures was added. Amino acids incorporated into aminoacyl-tRNA in 10 μL reaction mixtures are shown. Error bars indicate standard deviation of independent repeats of triplicate measurements.

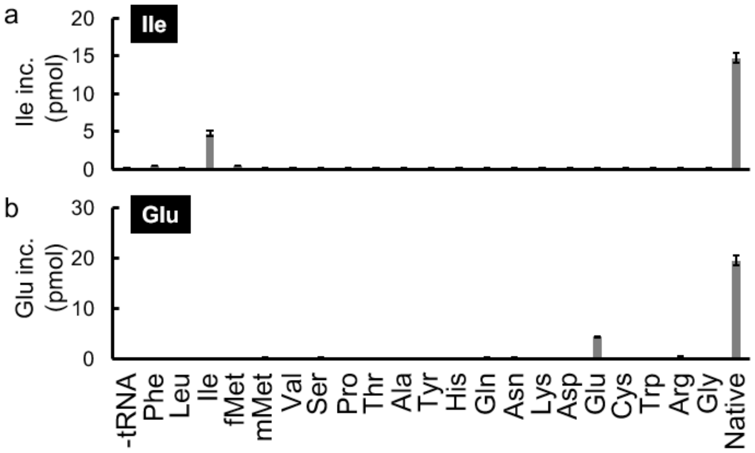

Supplementary Figure 3. Aminoacylation with increasing concentrations of aaRS. The amino acid acceptance of the prepared 21 iVTtRNAs was comprehensively analyzed with radioisotope-labeled Ile (a) and Glu (b). The concentration of each aaRS was 1.5 μM, and 2 A_{260} unit/mL of iVTtRNA (28–32 pmol in each 10 μL reaction mixture) or 40 A_{260} unit/mL of native tRNA mixtures was added. Amino acids incorporated into aminoacyl-tRNA in 10 μL reaction mixtures are shown. Error bars indicate standard deviation of independent repeats of triplicate measurements.

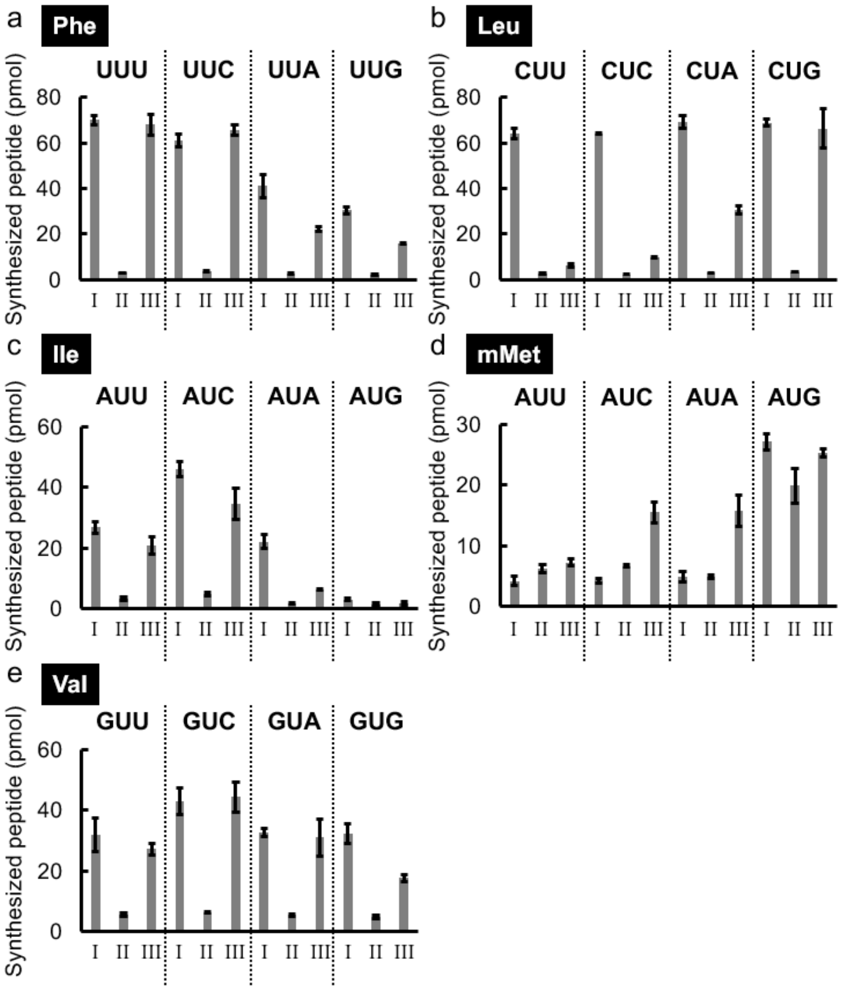

Supplementary Figure 4. Octapeptide synthesis analysis of the decoding of iVTtRNA$^{Phe}_{GAA}$, iVTtRNA$^{Leu}_{CAG}$, iVTtRNA$^{Ile}_{GAU}$, iVTtRNA$^{mMet}_{CAU}$, and iVTtRNA$^{Val}_{GAC}$. The yields of synthesized peptides in 50 μL reaction mixtures are shown. Reactions were performed with (I) native tRNA mixtures, (II) basal iVTtRNAs (**Supplementary Data 2**), and (III) basal and test iVTtRNAs for each test codon. Test iVTtRNAs were (a) iVTtRNA$^{Phe}_{GAA}$, (b) iVTtRNA$^{Leu}_{CAG}$, (c) iVTtRNA$^{Ile}_{GAU}$, (d) iVTtRNA$^{mMet}_{CAU}$, and (e) iVTtRNA$^{Val}_{GAC}$. Error bars indicate standard deviation of independent repeats of triplicate measurements.

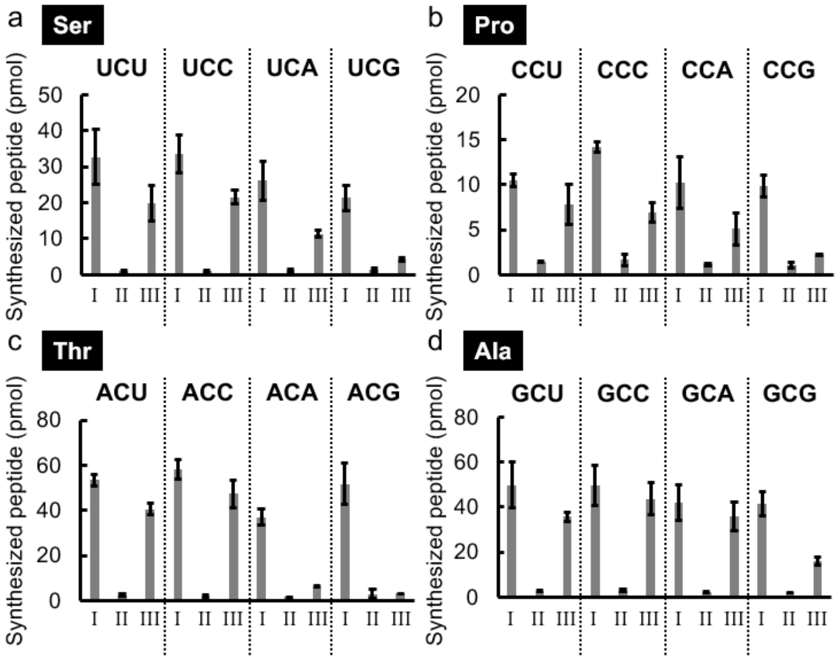

Supplementary Figure 5. Octapeptide synthesis analysis of the decoding of iVTtRNA$^{Ser}_{GGA}$, iVTtRNA$^{Pro}_{GGG}$, iVTtRNA$^{Thr}_{GGU}$, and iVTtRNA$^{Ala}_{GGC}$. The yields of synthesized peptides in 50 μL reaction mixtures are shown. Reactions were performed with (I) native tRNA mixtures, (II) basal iVTtRNAs (**Supplementary Data 2**), and (III) basal and test iVTtRNAs for each test codon. Test iVTtRNAs were (a) iVTtRNA$^{Ser}_{GGA}$, (b) iVTtRNA$^{Pro}_{GGG}$, (c) iVTtRNA$^{Thr}_{GGU}$, and (d) iVTtRNA$^{Ala}_{GGC}$. Error bars indicate standard deviation of independent repeats of triplicate measurements.

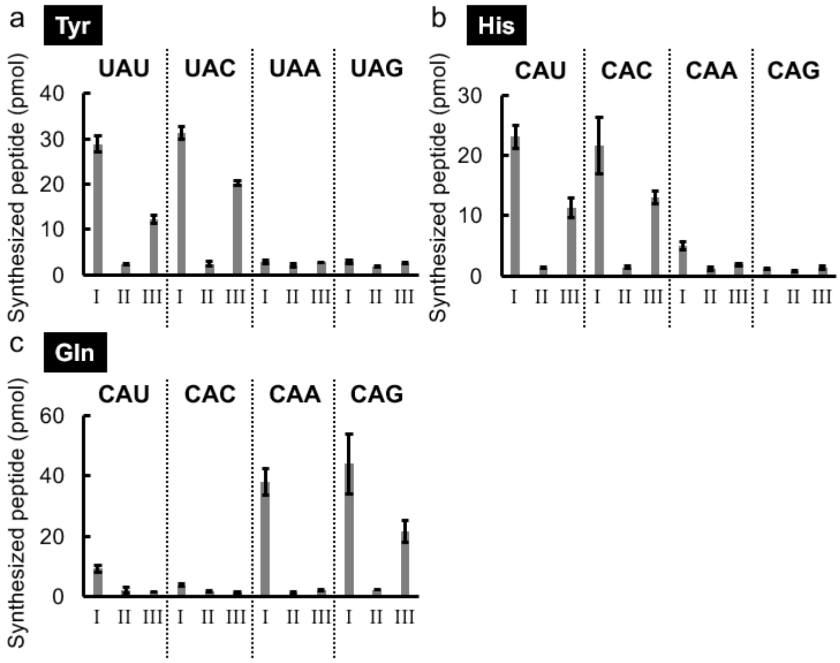

Supplementary Figure 6. Octapeptide synthesis analysis of the decoding of iVTtRNA$^{Tyr}_{GUA}$, iVTtRNA$^{His}_{GUG}$, and iVTtRNA$^{Gln}_{CUG}$. The yields of synthesized peptides in 50 μL reaction mixtures are shown. Reactions were performed with (I) native tRNA mixtures, (II) basal iVTtRNAs (**Supplementary Data 2**), and (III) basal and test iVTtRNAs for each test codon. Test iVTtRNAs were (a) iVTtRNA$^{Tyr}_{GUA}$, (b) iVTtRNA$^{His}_{GUG}$, and (c) iVTtRNA$^{Gln}_{CUG}$. Error bars indicate standard deviation of independent repeats of triplicate measurements.

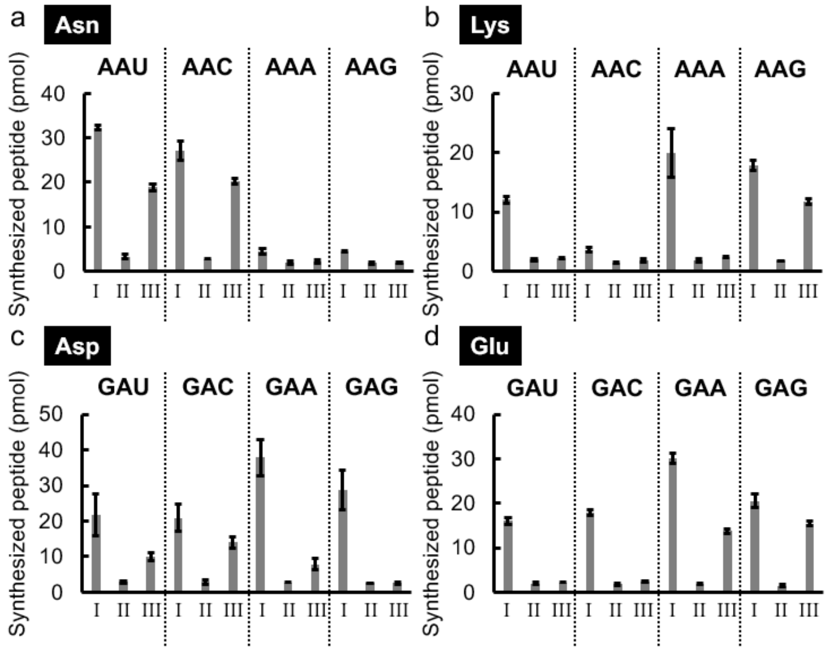

Supplementary Figure 7. Octapeptide synthesis analysis of the decoding of iVTtRNA$^{Asn}_{GUU}$, iVTtRNA$^{Lys}_{CUU}$, iVTtRNA$^{Asp}_{GUC}$, and iVTtRNA$^{Glu}_{CUC}$. The yields of synthesized peptides in 50 μL reaction mixtures are shown. Reactions were performed with (I) native tRNA mixtures, (II) basal iVTtRNAs (**Supplementary Data 2**), and (III) basal and test iVTtRNAs for each test codon. Test iVTtRNAs were (a) iVTtRNA$^{Asn}_{GUU}$, (b) iVTtRNA$^{Lys}_{CUU}$, (c) iVTtRNA$^{Asp}_{GUC}$, and (d) iVTtRNA$^{Glu}_{CUC}$. Error bars indicate standard deviation of independent repeats of triplicate measurements.

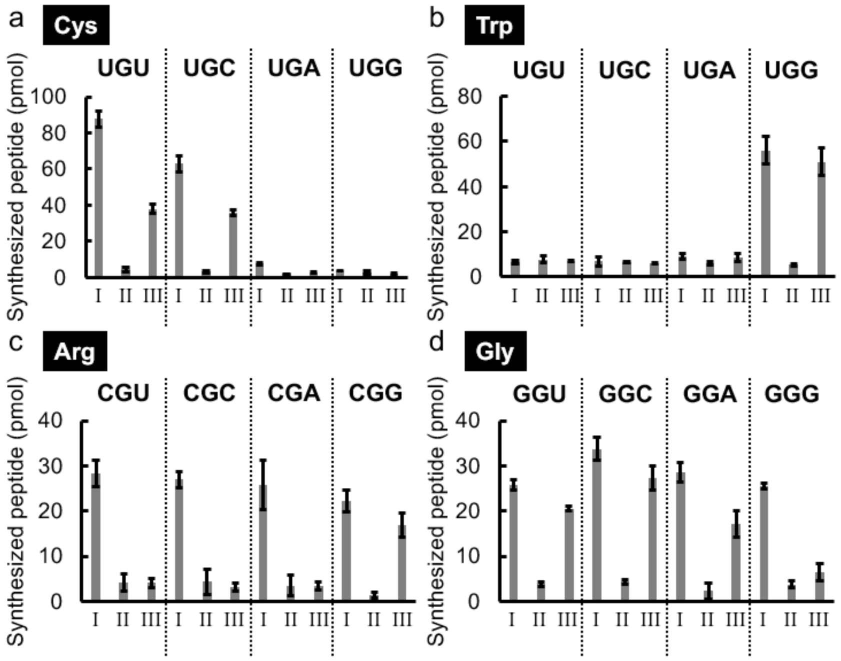

Supplementary Figure 8. Octapeptide synthesis analysis of the decoding of iVTtRNA$^{Cys}_{GCA}$, iVTtRNA$^{Trp}_{CCA}$, iVTtRNA$^{Arg}_{CCG}$, and iVTtRNA$^{Gly}_{GCC}$. The yields of synthesized peptides in 50 μL reaction mixtures are shown. Reactions were performed with (I) native tRNA mixtures, (II) basal iVTtRNAs (**Supplementary Data 2**), and (III) basal and test iVTtRNAs for each test codon. Test iVTtRNAs were (a) iVTtRNA$^{Cys}_{GCA}$, (b) iVTtRNA$^{Trp}_{CCA}$, (c) iVTtRNA$^{Arg}_{CCG}$, and (d) iVTtRNA$^{Gly}_{GCC}$. Error bars indicate standard deviation of independent repeats of triplicate measurements.

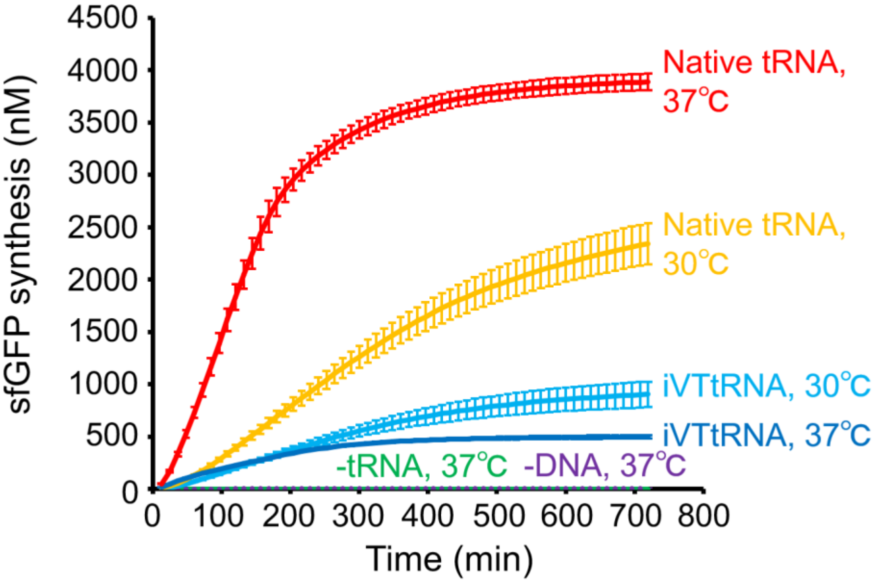

Supplementary Figure 9. Time-course analysis of sfGFP expression. Fluorescence of synthesized sfGFP was monitored. Blue and light blue lines represent a reaction with iVTtRNA mixtures at 37°C and 30°C, respectively. Red and yellow lines represent a reaction with native tRNA mixtures at 37°C and 30°C, respectively. Green dotted line represents a reaction without any tRNA at 37°C. Purple dotted line represents a reaction without any DNA template but with iVTtRNA mixtures at 37°C. Error bars indicate standard deviation of independent repeats of triplicate measurements. All experiments were performed with 60 A_{260} unit/mL iVTtRNA mixtures or 40 A_{260} unit/mL native tRNA mixtures.

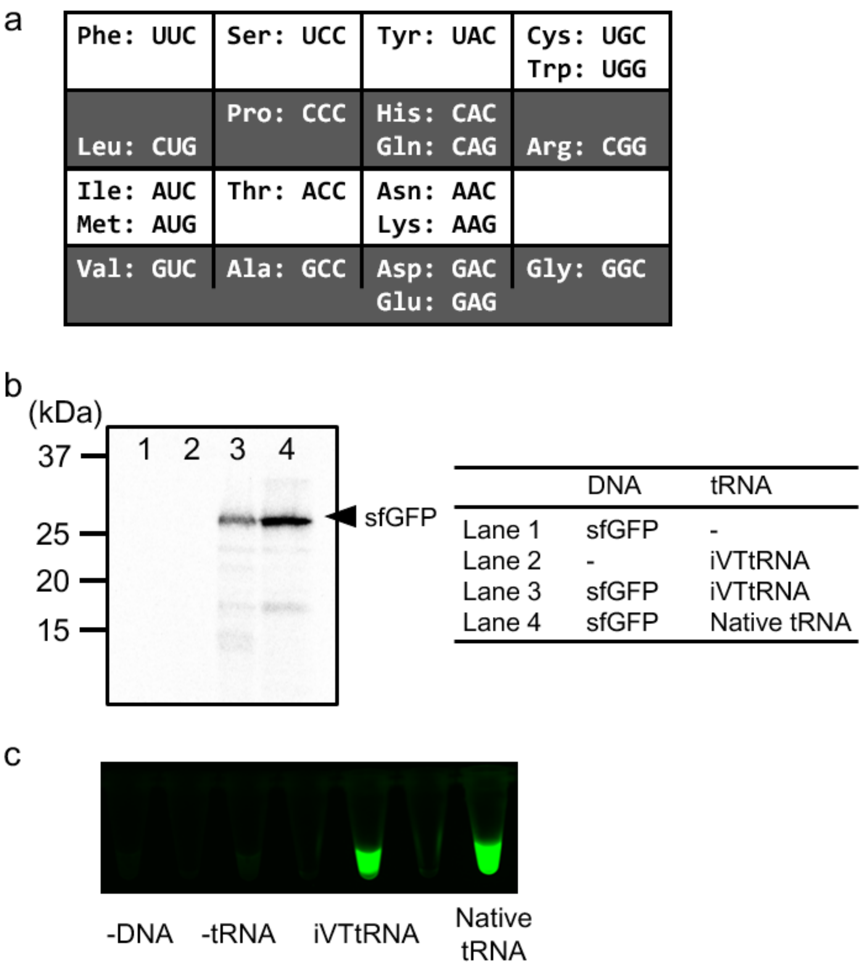

Supplementary Figure 10. sfGFP synthesis using iVTtRNAs without any wobble base pairs in the codon-anticodon interaction. (a) Genetic code table without any wobble base pairs. The DNA sequence for sfGFP expression was designed according to this table. (b) SDS-PAGE analysis of synthesized protein labeled with [^{35}S]Met. (c) Fluorescence images of synthesized sfGFP in reaction mixtures. All experiments were performed with 60 A_{260} unit/mL iVTtRNA mixtures or 40 A_{260} unit/mL native tRNA mixtures.

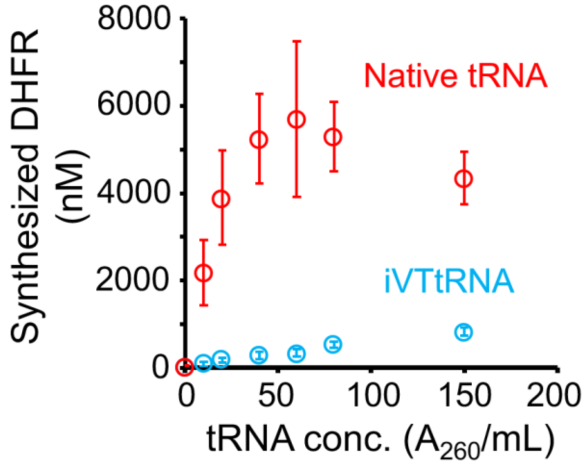

Supplementary Figure 11. Dependency of the yield of synthesized DHFR on tRNA concentration. Dependence of the yield of synthesized DHFR on the concentration of iVTtRNA mixtures, and native tRNA mixtures. Error bars indicate standard deviation of independent repeats of triplicate measurements.

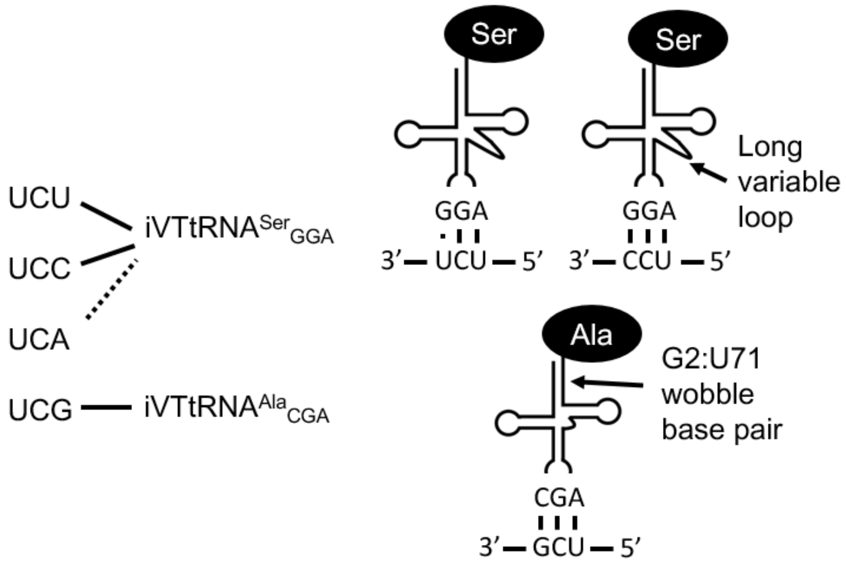

Supplementary Figure 12. Schematic diagram of genetic code redesigning using iVTtRNA$^{Ala}_{CGA}$. iVTtRNA$^{Ser}_{GGA}$, which has a long variable loop as a major identity element for recognition by seryl-tRNA synthetase, decodes UCU, UCC, and/or UCA codons, but struggles to decode the UCG codon (**Supplementary Fig. 5a**). Transplantation of the CGA anticodon into tRNA^{Ala} results in the formation of iVTtRNA$^{Ala}_{CGA}$ with a G2:U71 wobble base pair in the acceptor stem as a major identity element for recognition by alanyl-tRNA synthetase, which decodes the UCG codon as Ala. This facilitated separation of the Ser codon box into the Ser/Ala two-codon box.

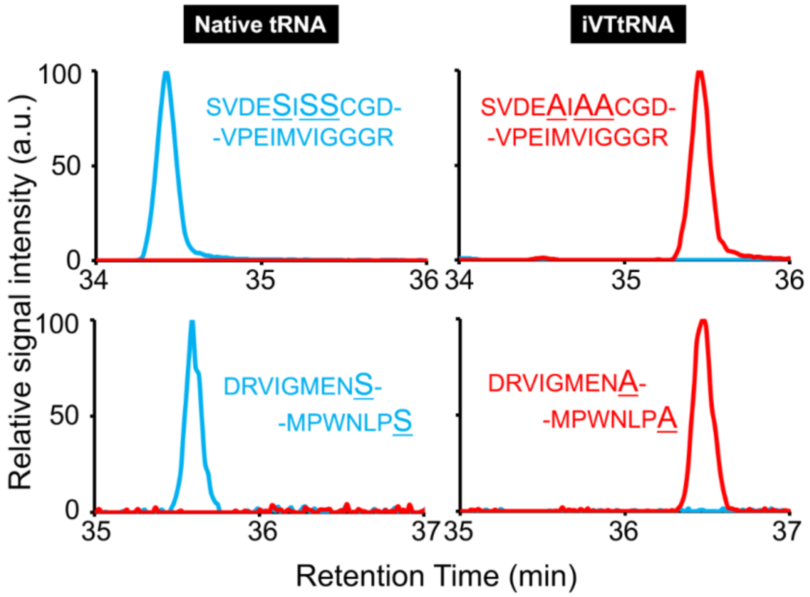

Supplementary Figure 13. LC-MS analysis of the synthesized proteins. Extracted ion chromatograms of ions with *m/z* = 1132.5303 (light blue) and *m/z* = 1108.5379 (red), corresponding to doubly charged ions of SVDESISSCGDVPEIMVIGGGR and SVDEAIAACGDVPEIMVIGGGR, respectively (upper panel) and *m/z* = 923.4347 (light blue) and *m/z* = 907.4398 (red), corresponding to doubly charged ions for DRVIGMENSMPWNLPS and DRVIGMENAMPWNLPA, respectively (lower panel) are shown. Sum of intensities within a window of twice the theoretical resolution (2xFWHM) at each retention time was normalized and plotted. Validity of each peak including MS/MS and retention time information was confirmed with Proteome Discoverer 2.2 software (Thermo Fisher Scientific, USA; **Supplementary Data 4**). Left panel shows the analysis of synthesized proteins using native tRNAs and right panel shows those using iVTtRNAs. Peptides in the upper panel were derived with tryptic digestion of the synthesized proteins and peptides in the lower panel were derived with Asp-N digestion of the synthesized proteins. The amino acids corresponding to the UCG codon are underlined. Ser was incorporated when native tRNAs were used and Ala was incorporated when iVTtRNAs were used.

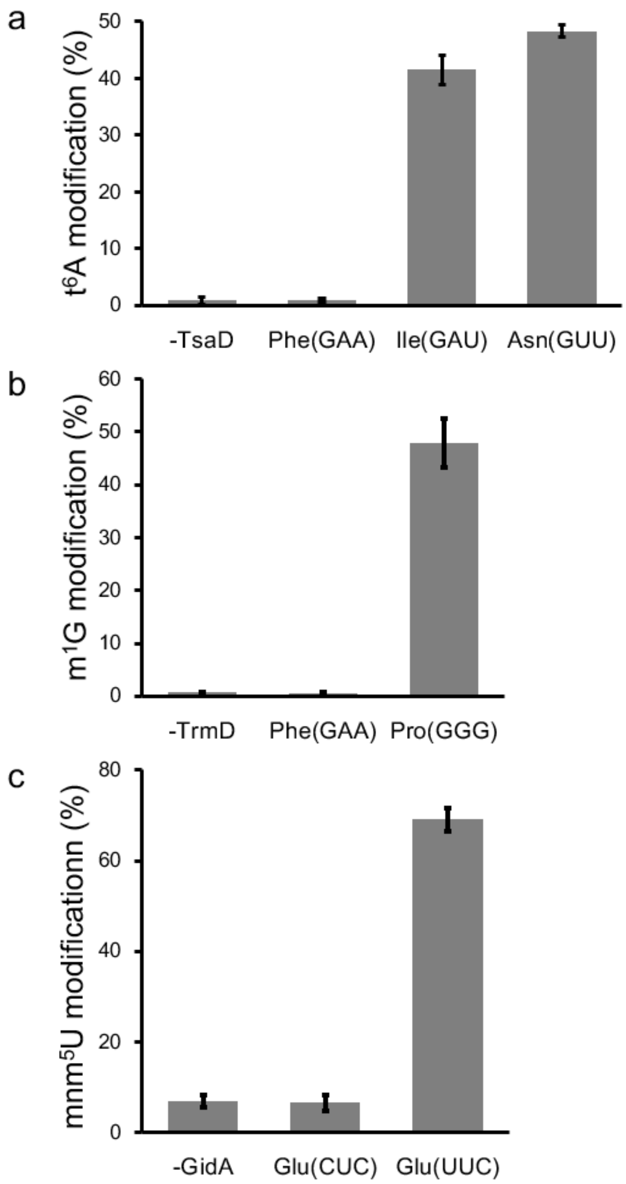

Supplementary Figure 14. Modification of iVTtRNAs. Efficiency of introducing (a) t^6A, (b) m^1G, and (c) mnm^5U into iVTtRNA was analyzed. (a) –TsaD represents a reaction without TsaD but with $iVTtRNA^{Ile}_{GAU}$. $iVTtRNA^{Phe}_{GAA}$ was used as a negative control that is not modified with t^6A. (b) –TrmD represents a rection without TrmD but with $iVTtRNA^{Pro}_{GGG}$. $iVTtRNA^{Phe}_{GAA}$ was used as a negative control that is not modified with m^1G. (c) -GidA represents a rection without GidA but with $iVTtRNA^{Glu}_{UUC}$ $iVTtRNA^{Glu}_{CUC}$ was used as a negative control that is not modified with mnm^5U. Error bars indicate standard deviation of independent repeats of triplicate measurements.

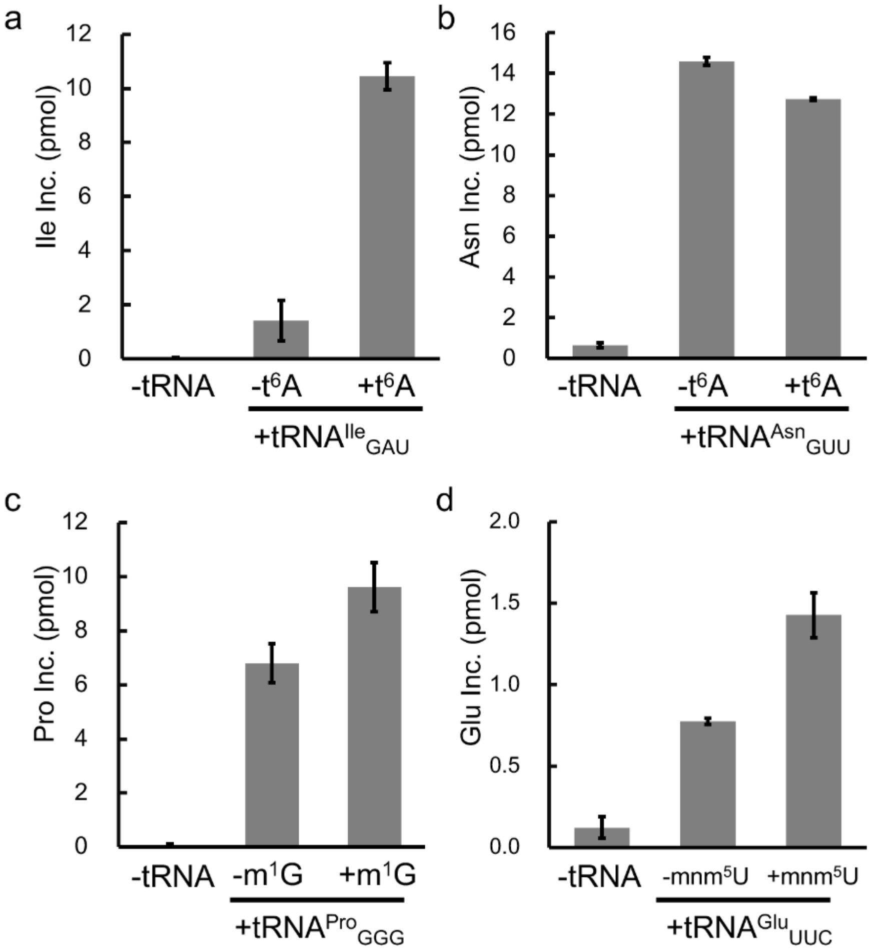

Supplementary Figure 15. Aminoacylation of modified iVTtRNAs. The amino acid acceptance of the unmodified or modified iVTtRNAs was analyzed. The concentration of each aaRS was 50 nM, and 2 A_{260} unit/mL of iVTtRNA (28–32 pmol in each 10 μL reaction mixture) was added. (a) $\text{iVTtRNA}^{\text{Ile}}_{\text{GAU}}$, (b) $\text{iVTtRNA}^{\text{Asn}}_{\text{GUU}}$, (c) $\text{iVTtRNA}^{\text{Pro}}_{\text{GGG}}$, and (d) $\text{iVTtRNA}^{\text{Glu}}_{\text{UUC}}$ were analyzed. Amino acids incorporated into aminoacyl-tRNA in 10 μL reaction mixtures are shown. Error bars indicate standard deviation of independent repeats of triplicate measurements.

Supplementary Notes

Supplementary Note 1. Detailed differences of the decoding properties

Several unusual results were observed in a series of octapeptide synthesis experiments. Invasion into non-canonical codons by tRNAs was observed for so-called two-codon boxes including Phe/Leu, Ile/Met, His/Gln, Asn/Lys, and Asp/Glu, even when using native tRNA mixtures, which may have resulted from the mis-translational activity intrinsically associated with the ribosome[1]. It appeared that Phe was incorporated into UUA and UUG codons, which are canonically Leu codons, in both cases using native tRNA mixtures and iVTtRNAs (**Supplementary Fig. 4a**). This might be due to the absence of Leu in reaction mixtures. Phe/Leu codons are representative near-cognate codons and it is known that Leu is incorporated by the Phe codons under the Phe starvation[2]. Inversely, the absence of Leu might facilitate the mis-incorporation of Phe by the UUA/UUG codons.

Similar results were found for His/Gln and Asn/Lys codon boxes. Synthesis of a small amount of peptide was detected with the CAA codon in the absence of Gln, and the CAU codon in the absence of His (**Supplementary Fig. 6b, c**), when native tRNA mixtures were used. Lys incorporation by the AAU codon (Asn) was also detected with native tRNA mixtures (**Supplementary Fig. 7b**), which is consistent with the previous result under Asn starvation[3]. However, these results were not observed when iVTtRNAs were used, suggesting that fully modified native tRNA mixtures are advantageous for such mis-incorporation in the absence of canonical amino acids. The use of iVTtRNAs with C at position 34 for Gln and Lys might also be beneficial for avoiding mis-incorporation.

While Ile incorporation results were reasonable for both native tRNA mixtures and iVTtRNAs (**Supplementary Fig. 4c**), complicated results were observed for Met incorporation using iVTtRNAs (**Supplementary Fig. 4d**). When AUG was used as the test codon, an appreciable amount of peptide was synthesized, even in the absence of $\text{iVTtRNA}^{\text{mMet}}_{\text{CAU}}$. This may suggest that $\text{iVTtRNA}^{\text{fMet}}_{\text{CAU}}$ compensates for the lack of $\text{iVTtRNA}^{\text{mMet}}_{\text{CAU}}$ for decoding the internal AUG codon. Although it was reported that $\text{iVTtRNA}^{\text{fMet}}_{\text{CAU}}$ has negative determinants that block its activity during elongation[4], addition of excess iVTtRNA might have resulted in non-canonical participation of $\text{iVTtRNA}^{\text{fMet}}_{\text{CAU}}$ during internal AUG decoding. The presence of $\text{iVTtRNA}^{\text{mMet}}_{\text{CAU}}$ resulted in AUC and AUA decoding with Met. This might be caused by a lack of N4-acetylcytidine modification at position 34 in $\text{iVTtRNA}^{\text{mMet}}_{\text{CAU}}$, which is required for decoding fidelity between AUA and AUG codons[5]. A lack of Ile and tRNA^{Ile} might

result in mis-incorporation at the AUC codon, in addition to AUA decoding. Although the activity of iVTtRNA$^{mMet}_{CAU}$ was barely detected when AUG was used for the test codon, presumably due to the presence of iVTtRNA$^{fMet}_{CAU}$, the decoding ability of the prepared iVTtRNA$^{mMet}_{CAU}$ was evident because addition of iVTtRNA$^{mMet}_{CAU}$ resulted in increased peptide synthesis when AUC and AUA were used as test codons.

We also observed complicated results for the Asp/Glu codon box, presumably due to the use of potassium glutamate in reaction mixtures and [^{14}C]Leu for labeling the synthesized peptide (**Supplementary Data 3**). When native tRNA mixtures were used, peptide synthesis was observed for all codons when the test amino acid was Asp (**Supplementary Fig. 7c**). This was due to the presence of both tRNA^{Glu} and Glu in reaction mixtures. The results of GAA and GAG decoding indicate canonical incorporation of Glu into the synthesized peptide. By contrast, GAG decoding was not observed with iVTtRNA$^{Asp}_{GUC}$, because tRNA^{Glu} was not included in reaction mixtures, while a small amount of Asp mis-incorporation was observed for GAA. It was strange that a reasonable amount of peptide was synthesized with native tRNA mixtures when test codons were GAU/GAC and the test amino acid was Glu. In this case, we did not include Asp in reaction mixtures, hence canonical incorporation of Asp cannot hypothetically occur (**Supplementary Data 3**). Although it cannot be concluded whether tRAN^{Glu} mistakenly decoded GAU/GAC codons in the absence of Asp, or a small but significant amount of Asp was carried over from the potassium glutamate salt, the main focus of this experiment was to examine the decoding ability of prepared iVTtRNAs, and separation of this codon box was possible if GAU/GAC codons were used for Asp and the GAG codon was used for Glu, as designed in this study (**Fig. 1a** and **Supplementary Fig. 1**).

Similar conclusions to those obtained for the Asp/Glu codon box may apply for the overall genetic code table. Separation of the Ile/Met codon box is possible when AUU is used for Ile and AUG is used for Met. The use of the CUG codon for Leu is helpful for Phe/Leu codon separation. Thus, the results of octapeptide synthesis experiments demonstrated that decoding fidelity can be maintained, even when iVTtRNA mixtures are used for the cell-free protein synthesis reactions.

Supplementary References

1. Mohler, K. & Ibba, M. Translational fidelity and mistranslation in the cellular response to stress. *Nat. Microbiol.* **2**, 17117 (2017).
2. Precup J., Ulrich, A. K., Roopnarine, O. & Parker, J. Context specific misreading of phenylalanine codons. *Mol. Gen. Genet.* **218**, 397-401 (1989).

3. Kramer, E. B. & Farabaugh, P. J. The frequency of translational misreading errors in *E. coli* is largely determined by tRNA competition. *RNA* **13**, 87-96 (2007).
4. Stortchevoi, A., Varshney, U. & RajBhandary, U. L. Common location of determinants in initiator transfer RNAs for initiator-elongator discrimination in bacteria and in eukaryotes. *J. Biol. Chem.* **278**, 17672-17679 (2003).
5. Taniguchi, T. *et al.* Acetate-dependent tRNA acetylation required for decoding fidelity in protein synthesis. *Nat. Chem. Biol.* **14**, 1010-1020 (2018).
