# Supplementary material for: Reconstituted cell-free protein synthesis using in vitro transcribed tRNAs
Source: Commun Biol. 2020 Jul 3;3:350. doi: 10.1038/s42003-020-1074-2 (PMC7334211; doi:10.1038/s42003-020-1074-2)
Supplement: Supplementary file 10 — Description of Additional Supplementary Files [file 42003_2020_1074_MOESM10_ESM.pdf]

Description of Additional Supplementary Files

File Name: Supplementary Data 1
Description: **Genes for each iVTtRNA used in this study.** The genes for each iVTtRNA was cloned into the vector and amplified using the listed DNA primers for *in vitro* transcription.

File Name: Supplementary Data 2
Description: **Summary of octapeptide synthesis analysis of the decoding ability of each iVTtRNA.** DNA templates for octapeptide synthesis were amplified using the listed DNA primers. Components in the peptide synthesis reaction mixtures, which depend on the iVTtRNA to be tested, are also listed.

File Name: Supplementary Data 3
Description: **DNA and amino acid sequences for protein expression.** DNA and amino acid sequences for DHFR and sfGFP expression are listed. 5'-UTR and 3'-UTR sequences, including T7 promoter and terminator sequences, are also listed.

File Name: Supplementary Data 4
Description: **LC-MS data.** Summary of LC-MS analysis of the synthesized proteins, which was derived with Proteome Discoverer 2.2 software (Thermo Fisher RIKEN BDR (Center for Biosystems Dynamics Research) 6-2-3, Furuedai, Suita, Osaka 565-0874 Japan Scientific, USA), is shown.

File Name: Supplementary Data 5
Description: Oligo DNAs for cloning of modification enzymes for specific iVTtRNAs.

File Name: Supplementary Data 6
Description: Source data for main figures.
